# Nutritional, Energy and Sanitary Aspects of Swine Manure and Carcass Co-digestion

**DOI:** 10.3389/fbioe.2020.00333

**Published:** 2020-04-29

**Authors:** Deisi Cristina Tápparo, Paula Rogovski, Rafael Dorighello Cadamuro, Doris Sobral Marques Souza, Charline Bonatto, Aline Frumi Camargo, Thamarys Scapini, Fábio Stefanski, André Amaral, Airton Kunz, Marta Hernández, Helen Treichel, David Rodríguez-Lázaro, Gislaine Fongaro

**Affiliations:** ^1^Western Paraná State University - UNIOESTE/CCET/PGEAGRI, Cascavel, Brazil; ^2^Embrapa Suínos e Aves, Concórdia, Brazil; ^3^Laboratory of Applied Virology, Department of Microbiology, Parasitology and Immunology, Federal University of Santa Catarina (UFSC), Florianópolis, Brazil; ^4^Department of Chemical and Food Engineering, Federal University of Santa Catarina, Florianópolis, Brazil; ^5^Laboratory of Microbiology and Bioprocesses, Federal University of Fronteira Sul, Erechim, Brazil; ^6^Laboratory of Molecular Biology and Microbiology, Instituto Tecnológico Agrario de Castilla y León, Valladolid, Spain; ^7^Division of Microbiology, Department of Biotechnology and Food Science, Universidad de Burgos, Burgos, Spain

**Keywords:** swine chain, biogas, biohydrogen, purification, nutrient

## Abstract

Renewable energy can assist the management of the effects of population growth and rapid economic development on the sustainability of animal husbandry. The primary aim of renewable energy is to minimize the use of fossil fuels via the creation of environmentally friendly energy products from depleted fossil fuels. Digesters that treat swine manure are extensively used in treatment systems; and inclusion of swine carcasses can increase the organic loading rate (OLR) thereby improving biogas yield and productivity on farms. However, the characteristics of the components including animal residues, proteins, lipids, remains of undigested feed items, antimicrobial drug residues, pathogenic microorganisms and nutrient contents, are complex and diverse. It is therefore necessary to manage the anaerobic process stability and digestate purification for subsequent use as fertilizer. Efficient methane recovery from residues rich in lipids is difficult because such residues are only slowly biodegradable. Pretreatment can promote solubilization of lipids and accelerate anaerobic digestion, and pretreatments can process the swine carcass before its introduction onto biodigesters. This review presents an overview of the anaerobic digestion of swine manure and carcasses. We analyze the characteristics of these residues, and we identify strategies to enhance biogas yield and process stability. We consider energy potential, co-digestion of swine manure and carcasses, physical, chemical, and biological pretreatment of biomass, sanitary aspects of swine manure and co-digestates and their recycling as fertilizers.

## Introduction

Meat is one of the most commonly consumed foods around the world, providing a good source of proteins and essential healthy nutrients and minerals, such as iron, zinc, amino acids, and group B vitamins (Wu et al., 2014). Meat consumption is linked to socio-economic factors, ethics and cultural (Font-i-Furnols and Guerrero, 2014). Swine production is a major part of the commercial livestock industry. Estimated world production exceeded 100 million tons in 2017 (USDA, 2019). China is the largest producer of swine meat (53.4 million tons in 2017) followed by the European Union (23.6 million tons), the US (11.6 million tons), and Brazil (3.7 million tons); these countries are thus responsible for approximately 83% of total global production (USDA, 2019).

There is a wide diversity of swine production systems throughout the world, and segregation is organized according to individual countries’ characteristics (FAO, 2018). Anaerobic digestion (AD) is a promising approach to residue management and energy production. It involves three steps: the breakdown of complex organic matter into soluble substances; the production of volatile fatty acids (VFAs) and hydrogen; and the conversion of VFAs and hydrogen to methane and CO_2_ (Wainaina et al., 2019).

Industrial swine production is characterized by intensive animal concentration generating large amounts of waste (47 L._HogBreeder_^–1^.d^–1^) and high energy demands. The waste is urine and feces mixed with wash water and food leftovers, pathogenic microorganisms, non-metabolized antibiotic drugs, organic matter, and nutrient contents (Kunz et al., 2009; Viancelli et al., 2012b). Several alternatives are available for the treatment of wastewater with high organic matter concentrations, including physiochemical treatment, aerobic processes (nitrification/denitrification), composting, and AD (Loyon, 2017). AD has become the most widely adopted due to the reduced costs of implementation, operation and maintenance (Deublein and Steinhauser, 2010).

In addition to the problem of the manure generated in the swine production chain, there is emerging concern regarding swine mortality. Swine inevitably die, and the carcasses are a major waste by-product of farming; safe disposal is indispensable to ensure environmental protection, as well as human and animal health (Wu L. et al., 2017). Carcasses may contain pathogenic microorganisms (viruses, bacteria and parasites), veterinary drug residues, and other chemical compounds (Zhong et al., 2017). Average swine mortality varies among major producers, and according to rearing method. In China, the mortality losses are 7, 10, 5, 1, and 1% in the sow, neonatal, nursery, growing, and finishing herds, respectively (Zhong et al., 2017). In the UK, these rates are 5.4% for sows, 2.8% for the growing phase and 2.7% for the finishing phase (AHDB, 2017). In Brazil, rates are: 7, 3.2, 4, 5.7, and 7% in neonatal, nursery, finishing, wean-to-finish and sow, respectively, similar to those of other countries (ABCS, 2014). There are various disposal methods, and AD is an emerging option that combines energy production and environmental protection. Many studies show that carcasses can be used to produce energy, and have a greater potential for producing hydrogen and methane (Tápparo et al., 2018; He et al., 2019) because of their high protein and lipid contents (Zhang and Ji, 2015).

## Nutrient and Energy Potential From Swine Chain Farm Residues

The methane production potential of swine manure depends on several factors, including organic matter concentrations, manure retention time inside barns and the growing phase (see Table 1; Gopalan et al., 2013; do Amaral et al., 2016; de Mito et al., 2018). The efficiency of biogas conversion into electric energy is function of the quality of the generator and swine manure characteristics, 1 m^3^ of swine manure in a finishing house unit can generate between 13.7 and 22.01 kwh. In Brazil, reported values are between 1.12 and 1.8 kwh per biogas meter cubic (Amaral et al., 2019).

**TABLE 1:** Biogas production per liter of swine manure obtained from various swine production processes.

Swine production arrangements	Effluent production (L_manure_.d^–1^)	Volatile solid concentration (kgVS.m^–3^)	Biogas yield (Nm^3^.kgVS^–1^)	m^3^_biogas_. m^–3^_manure_	Post-treatment options*
Farrow to feeder	45.52	15.08	0.560	12.99	Nitrification and
Farrow to wean	59.46	12.82	0.765	15.09	desnitrification.
Nursery house	1.39	3.8	0.970	5.70	Annamox Process,
Finish house	10.62	21.20	0.474	12.23	Desanonification.
Farrow to finish	94.75	19.25	0.375	11.10	

*For nutrients removal.

Such production of clean and renewable energy from swine manure significantly contributes to environmental protection, particularly with respect to the emission of methane, a greenhouse gas (GHG), and to the reduction of the demand for energy from fossil fuels. Cumulative emissions of GHGs from swine and manure at swine facilities are approximately 4.87 kg CO_2_ equivalents per kg of carcass; this could be reduced by up to 40% with improved manure management (Philippe and Nicks, 2015).

Environmentally friendly production and exploitation of methane requires maximum substrate utilization and minimum residual methane to reduce methane emissions (GHGs) from the digestate (Ruile et al., 2015). The type and composition of substrate, hydraulic retention time (HRT), organic loading rate (OLR) and process stability all contribute to outcome (Hernández and Rodríguez, 2013). The microbiological metabolic pathways may be classified into four successive steps: hydrolysis, acidogenesis, acetogenesis, and methanogenesis. In AD of swine manure, the methanogenesis stage is the most sensitive step due to the high biodegradability of the methane (Da Silva et al., 2015).

Digestates are mainly used for agricultural purposes: swine manure is biomass with value as a biofertilizer (Veroneze et al., 2019). Inadequate management can lead to the contamination of water courses, groundwater, soil and air, and these problems impede the sustainability and expansion of swine farming as an economic activity (Kunz et al., 2009). The quality of the digestate and its potential for agronomic use depends on several factors: the composition and variability of manure used for digestion (dependent on animal growing phase and nutrition system); biodigester technology; segregation and nutrient losses during substrate and/or digestate storage; efficiency of substrate pretreatment systems (solid separation after biodigestion) and digestate treatment; and the dilution of substrates and digestate with water (da Nicoloso et al., 2019). The nutrient content of swine manure is highly variable (Table 2), making laboratory analysis essential for fertilizer characterization. The laboratory results allow application of appropriate doses to fields, supplying the nutrient demand of the crops while avoiding excessive nutrient application and the consequent environmental impacts.

**TABLE 2:** Nutrient contents in swine manure from various sources and swine carcass used as a substrate for anaerobic digestion.

Source	Manure production		Nutrient			
	Volume	Unit	N	P	K	Unit
Feeder to finishing	1.64	m^3^.pig^–1^.y^–1^	8.0	4.3*	4.0**	kg.pig^–1^.y^–1^
Nursery house	0.94	m^3^.pig^–1^.y^–1^	0.4	0.25*	0.35**	kg.pig^–1^.y^–1^
Farrow to wean	8.32	m^3^.pig^–1^.y^–1^	25.7	18.0*	19.4**	kg.hog^–1^.y^–1^
Farrow to finishing	17.2	m^3^.pig^–1^.y^–1^	85.7	49.6*	46.9**	kg.hog^–1^.y^–1^
Swine carcass	–	–	3.1	0.61	0.3	kg. pig^–1^***

Adapted from da Nicoloso et al. (2019) and Rajagopal et al. (2014). *P_2_O_5_ form; **K_2_O form; ***considering one pig of 130 kg.

Tápparo et al. (2018) studied the biochemical methane potential of swine carcasses and report 1076 ± 48 L_Nbiogas_.kg_VSadd_^–1^ with a CH_4_ concentration of approximately 56%. This value indicates a biogas yield five times higher than that of swine manure. Considering one farm with 10,000 sows and mortality of 7%, the estimated potential of biogas production of 93,000 m^3^_biogas_ year^–1^, or around 52,000 m^3^ CH_4_ year^–1^. Likewise, Kirby et al. (2018) used dilution with water to digest swine carcasses with an OLR of 50 g_TS_ L^–1^ and 100 g_TS_ L^–1^ in a batch reactor giving biogas yields of 970 L_Nbiogas_.kg_VSadd_^–1^ and 850 L_Nbiogas_.kg_VSadd_^–1^, respectively. The acetic acid and propionic acid levels increased during the feeding phase and decreased during the final of non-feeding phase (approximately 30 days), but without significant inhibition of biogas production. For the start-up of a reactor, Kirby et al. used digestate taken from typical commercial mesophilic AD of food waste; such acclimatization can contribute to buffer capacity and stability of the process.

Mono-digestion methods are susceptible to the accumulation of VFAs and/or unionized ammonia, resulting in toxicity for methanogenic archaea and consequent reduction in methane production (Béline et al., 2017). One approach to avoiding this effect is co-digestion of two or more substrates. This offers several advantages: increased loading rate and consequent biogas production; improved buffer capacity and pH equilibration; better nutritional balance; dilution of inhibitory compounds; promotion of synergistic effects; and improved economy of biogas plants (Jiang et al., 2019). Many studies involved the use of various residues mixed with animal carcass, including sugar beet pulp (Kirby et al., 2018), vinasse (Dai et al., 2015), and manure (Tápparo et al., 2018).

## Co-Digestion of Swine Manure and Carcasses to Increase Bioenergy Production

AD is a versatile technology widely used to treat organic compounds in livestock production and for energy generation. Many substrates have been studied as co-substrates to improve biogas or hydrogen production from swine manure, including lignocellulosic residues (Neshat et al., 2017), food waste (Ye et al., 2013), microalgae (Wang et al., 2016), and animal carcasses (Tápparo et al., 2018). Swine manure is a good substrate for co-digestion process because of its alkalinity, good buffering capacity and the absence of the need for inoculation for reactor star-up.

Massé et al. (2008) investigated the psychrophilic AcoD of swine carcasses and swine manure in a sequence batch reactor (SBR) operated at 25°C. They found high values for biogas production and no inhibition (20 and 40 kg_carcass_ m^–3^_manure_ which are up to eight times commercial rates). Tápparo et al. (2018) studied the BMP of swine carcass and manure co-digestion in ratios of 3, 7.5, and 15 kg_carcass_m^–3^_manure_, and found biogas production of 52, 95, and 119% higher, respectively, than from swine manure alone. The methane yield increased 6% for each 1 Kg_carcass_.m^–3^_manure–add_; thus, co-digestion with carcass probably had a synergistic effect. However, at carcass loading simulating an emergency disease outbreak (117–467 kg_carcass_.m^–3^_manure_), an accumulation of VFAs in the AD system resulting in inhibition of biogas production. The greater swine carcass/manure ratio had a larger effect than did ammonia toxicity. For ratios of 117, 223, and 467 kg_carcass_.m^–3^_manure_, they report methane yields of 0.33 ± 0.03, 0.32 ± 0.05, and 0.24 ± 0.05 LCH_4_ g^–1^_CODfed_, respectively, and the methane compositions of 70% ± 2%, 68% ± 3%, and 66% + 7%, respectively (Rajagopal et al., 2014). Clearly, AD of swine carcasses is possible, combining a method to dispose of carcasses with increased biogas generation, but the process and operating criteria must be adapted to ensure complete residue degradation with gas production and material purification (Tápparo et al., 2018). More studies are needed to characterize anaerobic co-digestion with diverse ratios of swine carcasses to manure, in various reactor configurations and operational conditions (including HRT, temperature, and OLR).

### Inhibitors of Biogas and Biohydrogen Production From Swine Chain Residues

Many factors influence AD, and some intermediates and final compounds can play key roles in biogas production and inhibit the microorganisms involved. Table 3 summarizes the most prominent inhibitors and studies of swine manure and its co-digestion with carcasses.

**TABLE 3:** A summary of studies of inhibition of anaerobic co-digestion of swine carcass and other residues.

Substrate	Reactor design	Ratio	Organic loading rate	Temp. (°C)	pH	Free ammonia	Volatile fatty acids (g L^–1^)	References
Swine manure and swine carcass	Sequential batch	20 and 40 g_carcass_.L_manure_^–1^	3.2 g COD L^–1^ d^–1^	25	8.2	–	No accumulation	Massé et al., 2008
Swine manure and swine carcass	Sequential batch	117 g_carcass_.L_manure_^–1^	3.2 g COD L^–1^ d^–1^	25	7.95	302 ± 24	max 6	Rajagopal et al., 2014
		233 g_carcass_.L_manure_^–1^	3.2 g COD L^–1^ d^–1^	25	7.97	323 ± 12	max 5	
		467 g_carcass_.L_manure_^–1^	3.2 g COD L^–1^ d^–1^	25	7.82	313 ± 29	max 4	
Swine carcass	Batch	–	50 g_TS_ L^–1^	35	8.02	–	Increase during feed phase (to 5.9), decrease after 30 days of non-feeding phase (0.4)	Kirby et al., 2018
		–	100 g_TS_ L^–1^		7.99	–		
Swine carcass and sugar beet pulp	Batch	1:1 (TS base)	50 g_TS_ L^–1^	35	7.87	–	Increase during feed phase (to 5.1), decrease after 30 days of non-feeding phase (0.1)	
			100 g_TS_ L^–1^		7.95	–		
Swine carcass and vinasse	–	1:1 (VS base)	6.8 kg_VS_.m^–3^	35	7.75	600 mg L^–1^ (no effect on performance)	Accumulation of acids (reduction of 75% in biogas yield)	Dai et al., 2015
								

#### Ammonia Inhibition and Toxicity

Several authors describe the negative effects of high nitrogen concentrations on AD. FA concentrations exceeding 1,000 mg L^–1^ (6 g NH_4_–N L^–1^) in slaughterhouse waste digestion at 38°C and pH 8.1, are associated with ammonia inhibition (Lauterböck et al., 2012). Hejnfelt and Angelidaki (2009) reported that, during AD of separated or mixed swine by-products (meat and bone flour, fat, blood, hair, meat, ribs) at mesophilic and thermophilic temperature, high concentrations of ammonia (>7 g N dm^–3^) inhibited biogas production processes. Studies of swine carcass and manure co-digestion on the laboratory scale demonstrate an increase around 10 mg L^–1^ of NH_3_–N for each kg_carcass_ added per m^–3^_manure_, indicating that potential inhibition depends on the ratio used (Tápparo et al., 2019).

There is evidence that hydrogenotrophic methanogenic microorganisms are more tolerant than cetoclastic methanogenic species to for NH_4_Cl and urea nitrogen forms (Tian et al., 2018). Consistent with this, Yang et al. (2018) reported the enrichment of the hydrogenotrophic methanogenic *Methanobacterium* and *Methanoculleus* with increasing concentrations of NH_4_^+^-N, whereas counts of the acetoclastic methanogenic *Methanosaeta* were reduced at mesophilic temperatures.

Some studies investigated mitigation of the impact of high ammonia concentrations. One common strategy is co-digestion, where the feed mixture is further mixed with various nitrogen-deficient substrates to adjust the C:N ratio and decrease the nitrogen concentration below the inhibitory levels (Jiang et al., 2019). Furthermore, good buffering capacity of added substrate can mitigate the pH increase and contribute to lower ammonia toxicity from urea (Tian et al., 2018). Other strategies to prevent process failures include stripping, adaptation of the microbial community, and bioaugmentation (Krakat et al., 2017).

#### Acid Accumulation

The balance between the production and consumption of products produced by each AD step is determinant for successful biogas production, especially as concerns VFAs (Choong et al., 2016). During biogas production, there are two pathways to VFAs generation. In the first, monosaccharides and proteins are converted to short-chain acids including acetic, propionic, or butyric acid by acetogenic microorganisms. In the second, carbon dioxide and hydrogen are used as substrates for acetic acid production by acetogenic microorganisms, including homo-acetogenic bacteria – acetogenesis stages (Li et al., 2019). When VFAs production outstrips consumption, production is inhibited by decreasing pH. In a stable AD system, the concentration of VFAs is about 50–250 mg/L, and higher VFAs levels inhibit the system (Ren et al., 2018).

Co-inhibition by ammonia and VFAs has been described: methanogenic microorganisms were first inhibited by ammonia, resulting in increased VFA and hydrogen concentrations, and consequently higher toxicity in all AD steps (Tian et al., 2018). This led to a cycle in which there was VFAs accumulation, pH decrease, and thus inhibition by methanogenic products and accumulation of more VFAs. Common recommendations to minimize such inhibition and restore biogas production in commercial practice are to decrease ORL and to suspend feeding the reactor until biogas and hydrogen production are stabilized or, alternatively restarting the reactor. Both can cause significant economic losses. Nguyen et al. (2019) studied micro-aeration with alternatives to reduce VFAs concentrations and restore AD processes. Providing defined amounts of O_2_ allowed facultative heterotrophs rapidly (approximately 2 weeks) to consume excess VFAs accumulated during unstable periods.

#### Effect of Antimicrobials and Their Persistence in Digestates

Antibiotics are used in therapeutic or sub-therapeutic doses on animal farms, including swine farms, to prevent infections and treat disease (Yang et al., 2019). Spielmeyer (2018) reported that was possible to recover from <5 to 90%, depending on the antibiotic class, of the active substances in waste excreted by the animals because these compounds may not be completely metabolized. The most widely used antibiotics in animals include tetracyclines and sulfonamides, which are frequently detected in manure (Nurk et al., 2019). Antibiotics affect the complex microbial communities in biogas plants, and may improve or inhibit biogas production. Mustapha et al. (2016) observed that, in the presence of chloramphenicol, methanogenesis activity was lower, and biogas production was inhibited, whereas antibiotics had little effect on the hydrolysis, acidogenesis, and acetogenesis stages.

In batch assays of AD of swine manure, methane production was reduced by 56, 60, and 62% at oxytetracycline and chlortetracycline concentrations of 10, 50, and 100 mg L^–1^, respectively, but by the end of the experiment, the final concentrations of the antibiotics tended to be similar. Both compounds have affinity with solids in suspension, and consequently the fraction of antibiotics is lower in the liquid phase in ADs (Álvarez et al., 2010). Steinmetz et al. (2016), evaluated the persistence of tetracycline chlortetracycline, metacycline, and oxytetracycline (1.3–809 mg/L) in batch tests at mesophilic digestion (37°C). After 35 days, antibiotic concentrations had declined by between 46 and 98%. Nurk et al. (2019) reported similar results investigating the impact of the elimination of four common antibiotics (sulfadiazine, sulfamethazine, tetracycline, and chlortetracycline); rates of elimination were from 17 to 88%, with chlortetracycline showing the highest rates. Steinmetz and Gressler (2019) consider that AD is an important tool for eliminating antibiotics from livestock wastes, and thereby mitigate the risks associated with the use of drugs in concentrated animal feeding operations. Because substantial amounts of the antibiotics administered can be found in swine manure, there a significant putative effects on both biogas production and the prevalence of antimicrobial resistance genes. Further work is needed to understand the impact of additional swine carcasses in anaerobic digesters that treat swine manure.

## Pretreatments of Manure and Carcasses Biomass to Increase Biogas and Biohydrogen Yield

Various pretreatments have been studied recently for their ability to improve degradation of swine carcasses and manure and to increase biogas and biohydrogen production (Table 4). Such processes can improve the rate of hydrolysis in the digester, reducing the HRT in the digester; biogas plants can therefore be more compact (Bougrier et al., 2005; Carrere et al., 2016). Pretreat of manure and carcasses allows improvements in sanitary quality, reducing the pathogen load. Nevertheless, pretreatments can have negative effects on AD, and in some cases, decrease biogas production due to the formation of recalcitrant and inhibitory compounds (Carrere et al., 2016; Venturin et al., 2019).

**TABLE 4:** Pretreatments and effects on carcasses and swine manure biomass.

Pretreatment	Biomass	Positive aspects	Negative aspects	Conditions	Biogas and/or hydrogen yield	References
Alkaline	Manure	Easy retrieval (high volatility), not corrosive, low energy.	Implementation at a large scale, and chemical application.	Aqueous ammonia 32% w/w 20°C, 96 h.	244% increase in CH_4_ yield in 17 days of digestion.	Lymperatou et al., 2017
	Carcasses	No uncontrolled emission of gas, nutrients or pathogens into the environment. It is effective in eliminating pathogens.	Effluent production with toxic characteristics, high chemical and biological oxygen demand. Neutralization of digestate is necessary, i.e., mixing with other substrate.	Potassium hydroxide 2–8 M 20°C 20 days	600 mL CH_4_ g^–1^ VS in 42 days of digestion with KOH 2M.	Arias et al., 2018
Thermal	Manure	Promote the solubilization of cellulose and hemicellulose, increase in the total concentration of volatile fatty acids, and the hydrolysis of protein	High energy consumption	Continuously stirred tank reactors 70 ± 1°C, 1–4 days	281.6 mL CH_4_ g^–1^ VS in 22 days of digestion, with 3 days of pretreatment	Wu J. et al., 2017
	Carcasses	Effective pathogen inactivation. Decomposes organic matter in the solid phase.	Proteins are difficult to decompose, risking ammonium inhibition.	250 g carcasses 170°C, 30 min	236 mL CH_4_ g^–1^ VS in 25 days	Xu et al., 2018
Enzymatic	Manure	High conversion of carbohydrate and protein. Facilitates the acidogenesis step.	High mineral content and salinity (13 g L^–1^)	Stainless steel reactor 30–90°C Enzymes used: Delvolase; Delvozyme L; Filtrase NL; Bakezyme	36% increase in CH_4_ yield	Wang et al., 2015
	Carcass	Accelerate biomethane fermentation reaction	Accumulation of organic acids results in excessive acidification and slows the methane production rate	pH 6.5–9.0 Enzyme concentration (Porcine Trypsin) 0.5–2.5% 40°C, 24 h	104.59 mL CH_4_ L^–1^ of substrate in 23 days	He et al., 2019
Electrolysis cell	Manure	Electrolysis cell design is simple and can achieve high biofuel rates.	A large percentage of electrons are not successfully transferred to the current. Promising for the production of biohydrogen, but is not viable for biogas	Electrolysis cells: platinum cathode and graphite fiber anode, enriched with exoelogenic bacteria. 16–184 h 30°C. Current: 0.5 V.	14% increase in CH_4_ and 64% increase in H_2_	Wagner et al., 2009
Flocculation and Sieving	Manure	Remove the organic load and nutrients and simple to operate. Increases manure biodegradability.	Use of pretreatment chemical compounds that may affect the later stage of anaerobic digestion	Flocculation with commercial polymer (Chemifloc CV/300), and subsequent sieving (0.25 mm).	75.4% increase in CH_4_	González-Fernández et al., 2008; Wang D. et al., 2019; Wang L. et al., 2019
Grinding	Carcass	The digester needs to be emptied less frequently. Reduces the collection frequency and improves final product biosafety.	Not effective for pathogen control and features high power consumption.	13 mm and 4 mm double grinding	53.7% increase in CH_4_	Kirby et al., 2018
Ultrasound	Manure	Increases solubilization of organic matter, nitrogen and ammonia. It promotes particle disintegration, reduces bound protein and increases soluble protein.	High energy, reduces the efficiency of CH_4_ production, due to the formation of inhibitors	Ultrasonic probe (500 W, 20 kHz) Sonication pulses: 2 on 2 s 30°C	28% increase in CH_4_	Elbeshbishy et al., 2011
	Carcasses and manure (co-digestion)	Increases hydrolysis rate, methane production and inoculum methanogenic activity. Removal of volatile solids.	Release of flocculating agents and lignin compounds that decrease hydrolysis rate	Ultrasonic processor (30 kHz) 22 ± 5°C using specific energy inputs of 1000 kJ/kgTS 109 days	340 m^3^ CH_4_ t^–1^ VS	Luste et al., 2012
						

### Mechanical Pretreatments: Grinding and Sieving

Grinding and sieving are mechanical methods widely used before pretreatment and AD, especially of carcasses, aiming to reduce particle size (Kirby et al., 2018; He et al., 2019). The reduction particle size increases the surface area of the substrate, allowing greater contact with the substances used in pretreatment, improving the hydrolysis rate (Gwyther et al., 2011). The biomass that enters the bioreactor must be ground or milled; otherwise, the bioreactor and the process may malfunction. However, grinding and milling require a significantly more energy than other types of pretreatment (Barakat et al., 2013; Carrere et al., 2016).

### Thermal Pretreatments

AD of untreated residue and carcasses is not permitted under European Union legislation (1069/2009/CE) (Gwyther et al., 2011). These biomasses are required to be minimally sanitized (60 min, 70°C) or sterilized (20 min, 133°C, 3 bar) to ensure the sanitary quality of the digestate. In addition to pathogen inactivation and increased health safety, sanitization, and sterilization act as a thermal pretreatment, with two main effects: hydrolysis of organic matter present in the solid phase, including fatty acids, proteins, and carbohydrates; and structural loosening by pressure changes, leading to hemicellulose degradation and lignin transformation facilitating hydrolysis of cellulose and increasing the amount of biodegradable organic compounds in the digester (Luste et al., 2012; Carrere et al., 2016; Wu J. et al., 2017). Some of the carcass proteins are retained in the solids after thermal pretreatment, possibly causing ammonium inhibition in AD when proteins are decomposed. Furthermore, thermal pretreatment is very limited in practice because of the high energy consumption (Xu et al., 2018). The increase in biogas production needs to compensate for the energy expenditure required to pretreat the substrate if the system is to be economically viable.

### Physical Pretreatments: Electrolysis and Ultrasound

Electrolysis is used in pretreatments generating energy while treating wastewater. This method is based on the action of bacteria which oxidize organic matter such that free electrons generated from the biomass are transferred to the anode, and then, because of the charge difference, flow to the cathode. It requires a low voltage current to be applied to the effluent and then generates sufficient energy it become self-sustaining. It is possible to significantly reduce the organic charge via the exchange of electrons, and to obtain products of biotechnological interest such as biohydrogen and biogas (Min et al., 2005; Logan et al., 2007; Wagner et al., 2009).

Ultrasound is another effective pretreatment to improve methane generation. Ultrasound acts by forming cavitation bubbles in the liquid phase that collapse to a critical size, producing substantial heat and pressure at the liquid-gas interface, turbulence and shear in the medium (Bougrier et al., 2005; Luste et al., 2012). Consequently, ultrasound pretreatment solubilizes extracellular organic matter, increases chemical demand for soluble oxygen, causes disintegration and particle size reduction with consequent increased surface area; the hydrolysis rate is thereby increased and HRT decreases (Elbeshbishy et al., 2011).

### Chemical Pretreatments: Ammonia and Flocculation

Chemical pretreatment consists of the application of compounds such as acids, bases, and oxidants to improve biomass biodegradability and the production of biogas and biohydrogen. Ammonia is used for alkaline hydrolysis treatment of carcasses and swine manure. Although these are low-lignin feedstocks, and ammonia selectively attacks lignin, preserving carbohydrates that are precursors of the biogas (Gupta and Lee, 2010). The hydrolysis associated with ammonia may be due to the breakage of the bonds between lignin and hemicellulose by this alkaline agent and the delignification of biomass via the cross of the crystalline structure of cellulose (Jurado et al., 2013; Lymperatou et al., 2017). The ammonia can be recovered and recycled in the process or used for other purposes, including nitrogen fertilizers, making the technique economically viable as well as environmentally friendly (Lymperatou et al., 2017). Wang D. et al. (2019), Wang L. et al. (2019) proposed an integral system, including ammonia pre-treatment of residue with simultaneous recovery; ammonia striping was used as method with an efficiency as high as 99%, after ammonia crystallization, and could be reused for residue pre-treatment.

### Biological Pretreatments: Microorganisms and Enzymes

The time required for hydrolysis is shorter following enzyme-mediated biological pretreatment than for direct biological hydrolysis by anaerobic bacteria during AD (Wang et al., 2015). Because of the substantial amounts of proteins and lignocellulose in swine manure, enzymes such as protease, cellulase, and hemicellulase are used for hydrolysis and conversion of chemical oxygen demand (COD) to soluble forms. Coupling between pretreatment steps and enzymatic filtration can result in a less recalcitrant liquid, allowing increased AD efficiency (Wang et al., 2015). The presence in manure of residual antibiotics used during animal husbandry inhibits biogas production. Few biological pretreatment studies address the degradation of these compounds to increase the yield of biogas. The amounts of residual antibiotics in manure can be reduced during AD, but they cannot be completely eliminated (Yin et al., 2018). Pretreatments of swine manure with microorganisms or enzymes to increase biogas production are relatively scarce. However, β-lactamase exposed on the cell surface of *Escherichia coli* can eliminate β-lactam drug residues in dung solution in a short time (1 h), promoting the growth of metagenomic microorganisms during AD resulting in an up to 93.2% increase in methane production (Liu et al., 2019a).

## Sanitary Safety in Disposal of Animal Carcasses and Manure

The disposal of animal carcasses and manure requires attention, because they can vehicle diseases. This issue thus involves biosafety, public health, and environmental quality. Throughout history, the most widely used disposal method has been burial (carcasses) and spreading on soil (manure) because they are cheap and effective. However, the pathogens present in these materials must be contained or inactivated, to prevent the percolation of hazardous materials to water sources and other resources (Gwyther et al., 2011; Chowdhury et al., 2019). When the animal carcass is degraded without treatment (natural environment), pathogenic microorganisms can remain available in the soil and leach from it for long periods of time (Chowdhury et al., 2019).

Pretreatments of carcasses and manure biomasses can serve as environmental control procedures, reducing the biomass volume and its impact, allowing the reduction or even destruction of infectious material. Pretreatments must also respect the safety and integrity of the environment (Bot and Benites, 2005; Chowdhury et al., 2019). Biofertilizer based on swine manure and digestates (relative to other fertilizers, including chemicals, bio-organic and biofertilizers) acts against *Fusarium* wilt disease in bananas, probably due to reduction of *Fusarium* populations by increasing other microorganism counts (Shen et al., 2015). Tao et al. (2015). Analyses of microbial biomass, nitrogen, carbon, colony forming units of bacteria and fungi over 2 years led to the conclusion that all tested parameters were more favorable for organic fertilizers than synthetic fertilizers (Tao et al., 2015). Likewise, swine manure used as biofertilizer contributes to decreasing greenhouse gas emissions and increasing grain yields, illustrating how this strategy can diminish costs and ecological impacts (Topp et al., 2009; Hu et al., 2013; Kang et al., 2016).

Swine manure is rich in some metal ions, especially copper (Cu) and zinc (Zn) due to feed additives (Marcato et al., 2009; Legros et al., 2010; Alburquerque et al., 2012). Although Cu is an essential element for metabolic pathways and a component of structural and enzymatic proteins, it inhibits plant growth by modifying chromatin structure, photosynthetic and respiratory processes, membrane permeability, and other phenomena, probably via oxidative damage (Gratão et al., 2005; Yruela, 2005; Pilon et al., 2006). Zn decreases carbon assimilation by decreasing chlorophyll biosynthesis, and inhibits plant growth by causing leaf chlorosis and reduced rooting capacity (Castiglione et al., 2007; Chen et al., 2008; Dhir et al., 2008; Girotto et al., 2013). Zn may compromise membrane permeability and, consequently, the electron transport chain, and generate oxidative stress by interacting with the plant antioxidant defense system (de Magalhães et al., 2004; Gratão et al., 2005). Land disposal of swine digestate can lead to accumulation of metals, and in particular manganese (Mn), because Mn concentrations in digestates can be as high as 50–55 ppm, and concentrations as low as 1 ppm can be toxic for some crops (Bischofsberger et al., 2005). Mn is an important component of enzymes, for example the Mn-cluster of the oxygen-producing complex in photosystem II, and in manganese superoxide dismutase (Mn-SOD), essential for plant development (Lidon et al., 2004; Chen et al., 2015). However, excess Mn can compromise important structures and inhibit photosynthetic efficiency (Millaleo et al., 2013). It can lead to accumulation in vegetable products, a concern because of its effects on human health, mainly because of its capacity to cross the blood-brain barrier (Martinez-Finley et al., 2013).

Because of the repercussions for sustainability and health safety, an overall and multifaceted “One Health” strategy should be implemented to comply with the needs of environmental, human and animal health. This became feasible in the late 1990s through a formalized alliance between the World Health Organization (WHO), the FAO and the World Organization for Animal Health (OIE) (Nguyen-Viet et al., 2015). Animal manure and animal carcasses can carry pathogens that endanger humans, animals and the environment. Untreated or insufficiently treated animal manure may cause pathogens spread and foster infectious disease outbreaks (Wei et al., 2010; Elving et al., 2014). When pathogens are carried away by rainfall or irrigation, contamination may reach the water supplies or the food chain, allowing these microorganisms to cause disease far from the site of contamination (Elving et al., 2014). Few decontamination treatments currently applied to animal manure inactivate all types of pathogens. Most decontamination procedures, and the tests applied, are effective only against bacteria; while helminth eggs, protozoa cysts/oocysts, and enteric viruses are very resistant to environmental stresses often used in decontamination, such as increased temperature, pH variations and ultraviolet light.

Table 5 describes relevant examples of pathogens present in swine manure and the associated diseases, environmental survival and inactivation/treatment systems. A combination of factors such as temperature, pH, humidity, carbon content, nutrient availability, and antagonistic behavior can be adequate for animal manure treatment and sanitary improvement (Sidhu, 2001). Various methods are commonly used for microbiological risk reduction, and alkalizing chemicals and biological treatments are the most popular. Indeed, biological treatments are the most frequently employed, and in particular composting and AD are used to reduce pathogenic loads and to generate and store biogas. Anaerobic digesters are appropriate for pre-clarified effluents (Kunz et al., 2009).

**TABLE 5:** Main pathogens present in swine manure, their associated diseases, environmental survival, and inactivation/treatment systems.

**Pathogens**		**Main disease**	**Environmental matrices**	**Treatment system**	**References**
Bacteria	*Salmonella* spp.	Diarrhea, systemic disease and pneumonia in humans and animals (zoonotic)	Swine, bovine and poultry feces and manure	Anaerobic biodigester	Fongaro et al., 2014
	Methicillin-resistant *Staphylococcus aureus*	Swelling, warmth (zoonotic)	Swine, bovine and poultry feces and manure	Photochemical eradication by blue light activation of riboflavin	Slifierz et al., 2015
	*Listeria monocytogenes*	Listeriosis, encephalitis, abortions (zoonotic)	Swine, bovine and poultry feces and manure	_	Broes et al., 2019
Viruses	Rotavirus-A (RVA)	Diarrhea (zoonotic)	Swine and bovine manure	Membrane bioreactor Ultrafiltration with coagulation-sedimentation Ultraviolet radiation (UV)	Estes and Kapikian, 2007; Hmaied et al., 2015
	Porcine Circovirus type 2 (PCV2)	Multisystemic wasting syndrome	Swine manure	Aerobic tank; UASB	Chae, 2005; Viancelli et al., 2012a, b
	Porcine parvovirus (PPV)	Infertility and reproductive failure (not zoonotic)	Swine slurry	Peracetic acid 0.2% Moist heat 90°C	Mészáros et al., 2017
	Teschoviruses (TV)	Encephalomyelitis	Bovine and swine manure	–	Jimenez-Clavero et al., 2003; Haack et al., 2015
	Classical swine fever virus (CSFV)	Hemorrhagic infection	Swine slurry	Heat inactivation at 61°C	Turner et al., 2000; Becher and Thiel, 2001
Parasites	Ascaris suum	Diarrhea or gastroenteritis	Livestock waste	Ammonia and nitrogen	Maruyama et al., 1996; Vinnerås et al., 2003; WHO, 2006
	Cryptosporidium parvum and Giardia lamblia	Diarrhea or gastroenteritis	Livestock waste	Free ammonia Anaerobic digestion	Kinyua et al., 2016

Some pretreatment methods have potential for the control of pathogens in animal carcass biomass that is destined for subsequent use in anaerobic digesters for biogas production. Positive effects can be found for alkaline hydrolysis, with which it is possible to eliminate pathogens completely, including the agents of transmissible spongiform encephalopathy. The mechanism may involve the action of the alkaline agent and warming that break microbial cell walls. In addition, this procedure does not produce greenhouse gas emissions (Nutsch and Spire, 2004). Thermal treatment efficiently controls a wide range of pathogens; *Enterococcus faecalis*, *Salmonella* Senftenberg, Parvovirus, helminth eggs and other pathogens can be eliminated by heating (Liu et al., 2019b). Finally, emerging non-thermal technologies can contribute to pathogen control: pulsed electric fields (PEF), microwaves, pressurization, ultrasound and chemical treatments. PEF considerably reduced *Enterococcus faecalis* and *E. coli* counts (by 0.5 up to 3.5 log_10_) in animal waste (Liu et al., 2019b, c).

### Technic-Economic Advantages

From an economical perspective, swine manure treatment system can represent a significant portion of the investment on a swine production facility, but can also generate an added value by providing farm revenues through its by-products, such as carbon credits, fertilizer and biogas; estimations can range from an internal rate return (IRR) 6.4–28.4% per year (Kunz et al., 2009). It can depend on the project’s objectives and scale, but matches with this technology characteristic, since the focus is high scale CAFOs, where are limited crop areas for manure disposal. The main advantages associated with the biogas treatment is to increase the supply of alternative sources of electric energy and the effective treatment of waste produced by farms (Freitas et al., 2019). In a study analyzing the life cycle assessment of swine production of four manure management systems, the authors concluded that that the use f a biodigester for energy purposes shows the best environmental performance for almost all the environmental impacts, mainly due to the biogas recovery and the potential of energy saves (Cherubini et al., 2015). Consequently, the implementation of efficient treatment systems can promote social benefits due to a protection of animal and human health, while also can reduce environmental problems such as excessive waste disposal in the soil, contamination of groundwater and eutrophication of water bodies (Freitas et al., 2019).

## Final Considerations

The swine production chain is of enormous importance to nutrition worldwide, but has numerous associated issues, including those relating to protein production, food demands, energy needs and balance in humans, animal health, and environmental concerns. Residues from this chain can be treated and used for biofuel purposes. Co-digestion of swine manure and carcass biomass generates biogas and bio-hydrogen; the resulting digestate is rich in nutrients and is valuable for use in farming and nutritional recycling. Thus, appropriate management would make a large contribution toward sustainability.

## Author Contributions

DT and PR prepared the first draft of the manuscript, that was firstly revised by GF. RC, DM, CB, AF, TS, FS, AA, AK, MH, and HT contributed with inputs in the different sections of the manuscript. DR-L and GF revised the drafts and prepared the final version of the manuscript.

## Conflict of Interest

The authors declare that the research was conducted in the absence of any commercial or financial relationships that could be construed as a potential conflict of interest.
